# Serum Thymidine Kinase Activity: Analytical Performance, Age-Related Reference Ranges and Validation in Chronic Lymphocytic Leukemia

**DOI:** 10.1371/journal.pone.0091647

**Published:** 2014-03-12

**Authors:** Eszter Szánthó, Harjit Pal Bhattoa, Mária Csobán, Péter Antal-Szalmás, Anikó Újfalusi, János Kappelmayer, Zsuzsanna Hevessy

**Affiliations:** Department of Laboratory Medicine, Medical and Health Science Center, University of Debrecen, Debrecen, Hungary; University of Pecs Medical School, Hungary

## Abstract

**Background:**

To date no age-related reference ranges are available for serum thymidine kinase (TK1) activity. Being a proliferation marker, it may be used as a prognostic marker in malignant diseases, including chronic lymphocytic leukemia (CLL). Our aim was to establish age-specific reference ranges for TK1 and examine its utility as a screening marker in CLL, a disease of the elderly.

**Methods:**

Serum TK1 activity was measured by a competitive chemiluminescent immunoassay in 369 healthy adults and 115 de novo CLL patients.

**Results:**

We observed a statistically significant decline in TK1 activity from young (18–35 years) to middle-aged (36–60 years) and further on to elderly (60–86 years) healthy individuals. Age-related reference range was: <30 U/L for young, <25 U/L for middle-aged and <19 U/L for elderly. There was no difference in TK1 activity between the studied healthy men and women. In CLL patients, TK1 activity was the highest in the advanced Rai stages. The area under the receiver operating characteristic curve (ROC-AUC) for TK1 was 0.840 (95% CI: 0.787–0.892), for differentiating CLL patients from age and sex matched healthy controls, with a cut-off value of 10.5 U/L (sensitivity: 80.9%, specificity: 73.4%). TK1 was significantly elevated in CD38+/Zap70+ CLL patients, and showed significant correlation with WBC and absolute B-cell count.

**Conclusion:**

In the healthy, serum TK1 activity does not differ in the two sexes but declines significantly with age. As such, use of age-related reference ranges is warranted, especially when evaluating CLL patients who generally belong to the elderly age group.

## Introduction

Distinguishing from a few tumor specific biomarkers (PSA, AFP, HCG etc) [1]–[3], thymidine kinase 1 (TK1, ATP; thymidine 5′-phosphotransferase; EC.2.7.1.21) is one of the non-specific tumor markers that can be measured in continuously dividing cells [4]. TK1 is a key cellular enzyme in the so-called salvage pathway of DNA synthesis. It phosphorylates thymidine into thymidine-monophosphate. The enzyme has two isoforms, TK1 is found in the cytoplasm, while TK2 remains in the mitochondria. The activity of TK1 is cell cycle-dependent, its level begins to rise in G1/S phase and reaches its peak in the G2/M phase [5]–[9].

TK1 can be isolated from many different cell and tissue types, and elevated serum TK1 activity is documented in many types of cancer, nonetheless, the mechanism of TK entering the systemic circulation is not well understood [10]–[13]. Furthermore, TK has been reported as a prognostic marker in chronic lymphocytic leukemia (CLL) and in non-Hodgkin’s lymphoma [14]–[17]. In the last decade several studies showed the prognostic value of CD38 and Zap70 expression in CLL [18]–[22]. Elevated serum TK1 level was found in patients with unmutated IGHV gene, and in those with higher expression levels of Zap70 and CD38, as well as advanced disease stage [23]–[24]. Konoplev et al showed that high serum TK1 levels predict poorer overall survival and higher risk of developing large B-cell lymphoma (Richter syndrome) [16]. The first TK measurement method was a radio-enzymatic assay using ^125^I-deoxyuridine [25]; later a non-radiometric, competitive, enzyme-linked, immunosorbent assay (ELISA) was developed [26]. Wu et al developed a method for measuring not the activity but the concentration of TK in serum [27]. Most recently, a liquid chromatography – tandem mass spectrometry method was described by Faria et al [28]. Nisman et al compared two commercially available immunoassays in breast cancer patients, and despite reporting differences between the two assays, concluded that both assays were adequate for tumor recurrence prediction [29].

Hallek et al found that a serum TK level of 7.1 U/L was the cutoff value that could differentiate shorter (high TK levels) and longer (low TK levels) progression-free survival subgroups in Binet A stage disease [15]. In the French-German CLL7 trial, one of the adverse risk factors to identify high-risk Binet A patients was a serum TK level >10 U/L [30], [34]. Magnac et al examined the relationship between serum TK levels and IGHV mutational status and found that TK levels above 15 U/L correlated well with the IGHV unmutated status of the patients [31]. A thorough review on prognostic markers of CLL was published by Sagatys & Zhang in 2012, encouraging the establishment of a universal algorithm to improve prognostic accuracy [32]. Molica et al carried out a comparative validation analysis of the MD Anderson Cancer Center (MDACC) [33] and the modified German [34] CLL score. They found that for any novel prognostic scheme, these two scores should be the benchmark for comparison [35]. In a recent paper, Molica suggested building an international, comprehensive CLL prognostic index [36].

The primary aim of this study was to establish age-related reference ranges for serum TK. Our hypothesis was that healthy older individuals might have lower TK activity as compared to younger adults. In addition, in a cross-sectional, case-control study, we measured serum TK activity in *de novo* CLL patients who were still therapy naïve, and in an equal number of age- and sex- matched healthy volunteers. Knowing that CLL is a disease prevalent in the elderly, the utility of the age-specific reference range was examined in the studied CLL cohort.

In the CLL patients, we compared the serum TK activity with other laboratory parameters, such as white blood cell count, absolute B cell count, CD38 and Zap70 expression.

## Patients and Methods

### Study population, healthy cohort for establishing reference ranges

**Figure 1:**
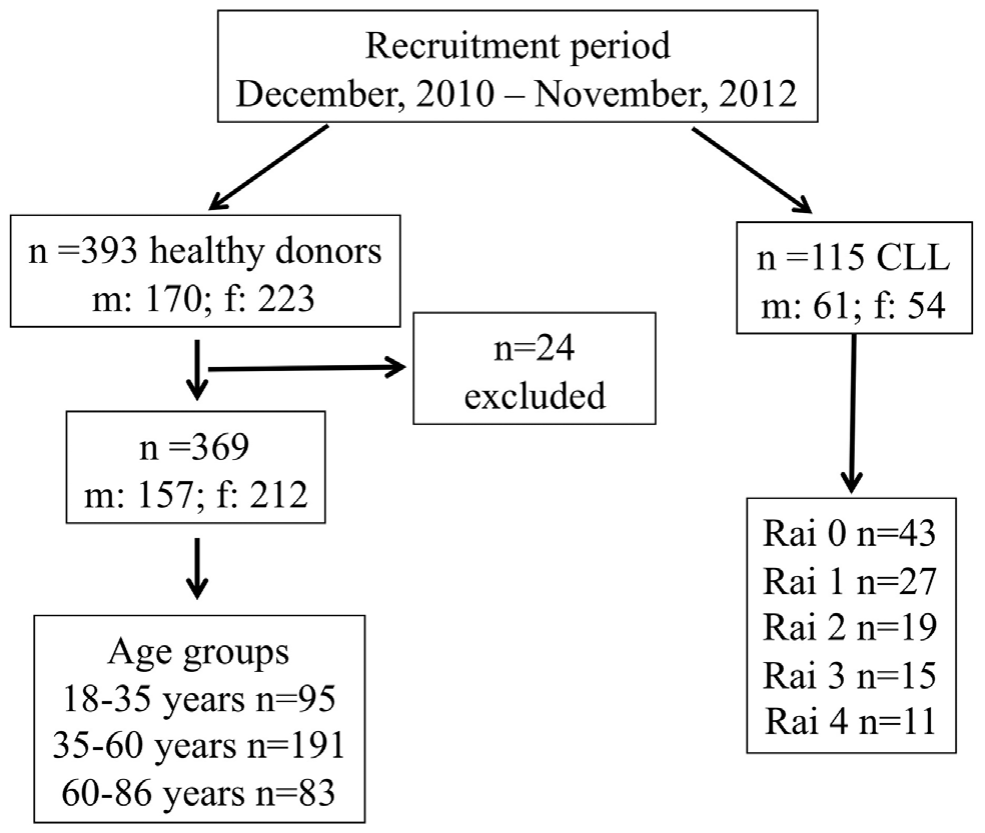
Flow diagram of study population.

Healthy volunteers (n = 369) between 18 and 86 years of age (median: 45 years, men: 157, women: 212) were recruited from blood donors and staff of the faculty with values ≤2× upper limit of normal for all measured laboratory parameters. Exclusion criteria were cancer, metabolic and inflammatory diseases. Due to non-confirmation with selection criteria data from 24 individuals was excluded from the final statistical analysis. To examine the influence of age, the reference population was subgrouped into the following age groups: 18–35 years (young), 35–60 years (middle-aged) and 60–86 years (elderly) (Figure 1).

### Study population, CLL cohort

Patients presenting with *de novo* CLL between December, 2010 and November, 2012 were included in this cross-sectional, analyst-blinded, age and sex matched case-control study (n = 115). The mean age (range) of the individuals in both groups was 65.6 (46–86) years, and the male:female ratio was ∼ 1:1 (61:54). As per Rai staging, 43 were in stage Rai 0, 27 were in Rai 1, 19 were in Rai 2, 15 were in Rai 3 and 11 were in Rai 4 (Figure 1). In this cohort, 29 and 86 patients belonged to the middle-aged and elderly age-groups, respectively.

This study was approved by the Ethics Committee of the University of Debrecen in accordance with the Declaration of Helsinki. All participants gave written informed consents.

### Laboratory methods

#### Measurement of thymidine kinase activity

TK1 activity was measured by an indirect, modified, two-step competitive chemiluminescent immunoassay on a Liaison immunochemistry analyzer (DiaSorin S.p.A., Saluggia, Italy). The calibration curve is defined mathematically by the manufacturer (master curve), which includes the pathologic values as well. For quality control the reagent kit includes two QC samples (low and high). In order to evaluate assay performance, accuracy and precision were determined from QC samples in low and high range (QC1 and QC2, respectively). Precision was expressed in CV% and accuracy was assessed as percent deviation from the nominal activity. Intra-assay precision and accuracy were determined from 9 replicates of each QC sample on a single occasion while inter-assay precision and accuracy were determined by analyzing 18 different operating days. The results were analyzed as a linear regression of the nominal vs. observed values. Linearity was evaluated by serial dilution of a patient sample with markedly elevated TK activity.

**Determination of CD38 and Zap70 positivity of CLL cells by flow cytometry**

White blood cell counts were measured by an Advia 120 hematology analyzer (Siemens AG, Erlangen, Germany). Absolute B-cell count was calculated from the percentage of CD19+ cells and white blood cell count.

Flow cytometric measurements were carried out on FACSCanto II flow cytometer, the results were evaluated by FACSDiva software (BD Biosciences, San José, CA, USA). The antibodies used were: CD19-PE-Cy7 (clone: J3-119, Beckman-Coulter, Brea, CA, USA); CD38-APC (clone: HB7, BD Biosciences) and Zap70 (unlabeled, clone: 2F3.2, Merck-Millipore (Upstate), Billerica, MA, USA). The patient was considered CD38 and/or Zap70 positive if ≥20% of the CLL cells expressed the respective markers.

#### Fluorescence *in situ* hybridization (FISH)

Interphase fluorescence *in situ* hybridization (FISH) was performed on cell suspension originated from peripheral blood according to standard protocol. Cells were obtained by 72 hour culture in the presence of 12-O-tetradecanoylphorbol-13-acetate (TPA) (Sigma-Aldrich, Munich, Germany) or by direct preparation from uncultured peripheral blood. The probes used for FISH analysis included LSI Rb1(13q14)/13q34, LSI p53, LSI ATM (Abbott Molecular Inc, Des Plaines, USA). Cells were counterstained with DAPI (4,6-diamidino-2-phenylindole). At least 200 interphase cells were analysed for each probe in each case. The images were captured by Zeiss Axioplan2 (Carl Zeiss, Zaventem, Brussels) fluorescence microscope and analysed by ISIS software (MetaSystems, Altlussheim, Germany).

### Statistical Analyses

Kolmogorov-Smirnov test was used for the evaluation of the normality of the data. Most parameters were non-normally distributed; therefore analyses were performed by Mann-Whitney U test. The Spearman’s rho was calculated for correlation analysis. P<0.05 was regarded as statistically significant. All analyses, including drawing of receiver operating characteristic (ROC) curves and estimation of the area under the ROC curve (AUC), were performed using the SPSS Statistics software, version 19.0 (IBM Corps., Armonk, NY, USA).

## Results

Reproducibility and accuracy tests gave excellent results. The nominal mean TK1 activity of QC1 was 14.8 U/L. Intra-assay measured mean was 15.0±1.58 U/L, precision CV of QC1 was 10.54%, accuracy was 101%. Inter-assay measured mean of QC1 was 14.8±2.23 U/L, precision CV was 15.03% and accuracy was 100%. The nominal mean activity of QC2 was 53.7 U/L, intra-assay measured mean was 62.4±6.34 U/L, precision CV was 10.16% and accuracy was 116%. Inter-assay measured mean of QC2 was 49.15±6.27 U/L, precision CV was 12.76% and accuracy was 92% (Table 1). The test was linear from 0.5 U/L to 100 U/L.

**Table 1:** Inter-assay and Intra-assay precision and accuracy for serum thymidine kinase activity of QC1 and QC2 samples.

	Nominal concentration (U/L)	
	14.8 (QC1)	53.7 (QC2)
Observed concentration (U/L)		
Intra-assay mean ± SD	15.0±1.58	62.4±6.34
Intra-assay precision (%CV)	10.54	10.16
Intra-assay accuracy (%DFN)	1.35	16
Inter-assay mean ± SD	14.8±2.23	49.15±6.27
Inter-assay precision (%CV)	15.03	12.76
Inter-assay accuracy (%DFN)	0	–8.5

SD, standard deviation; %CV, percent coefficient of variation; %DFN, percent deviation from nominal value.

**Figure 2:**
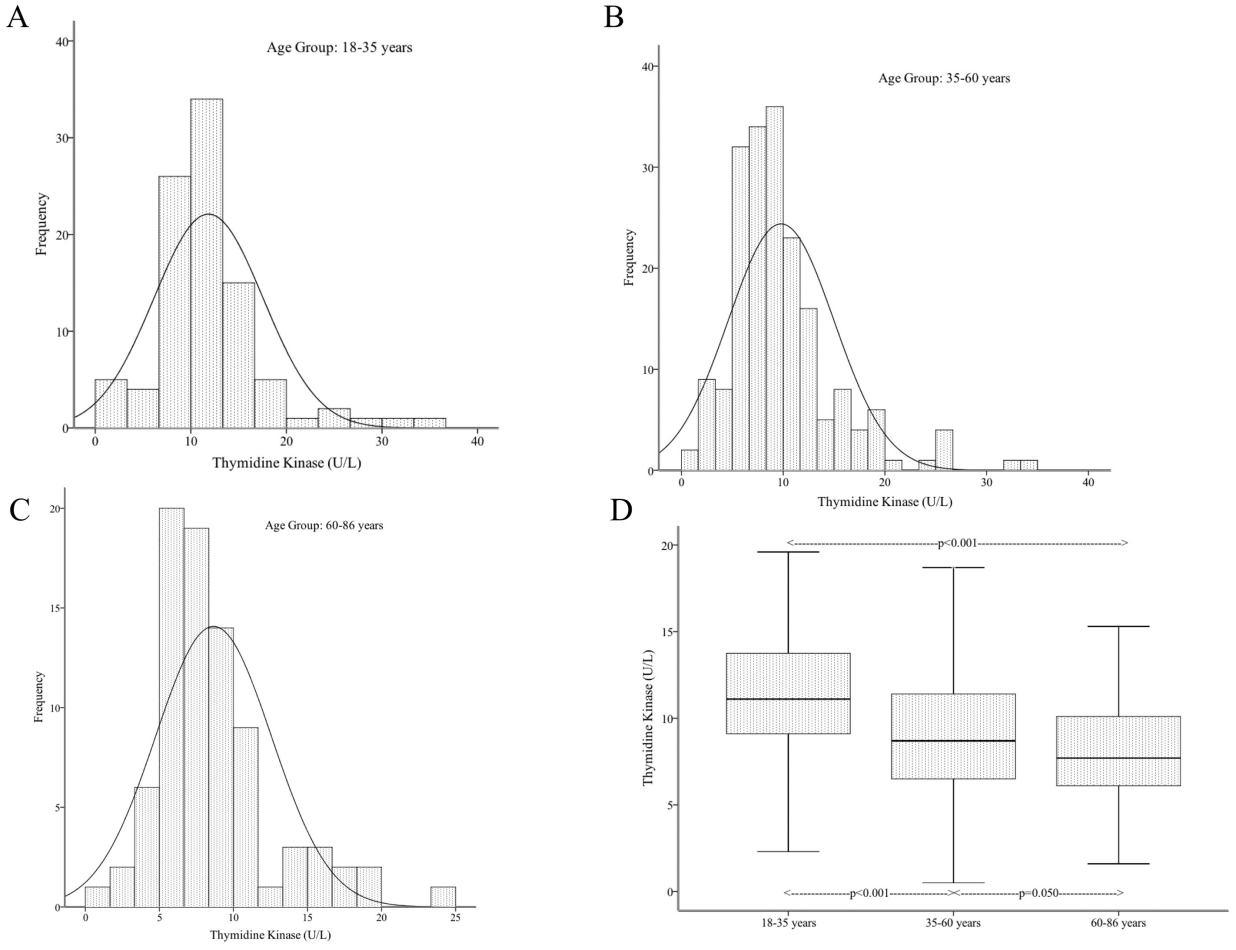
Age-related frequency distributions of serum thymidine kinase activity in age groups 18–35 years (A), 35–60 years (B) and 60–86 years (C). Box and whisker plots showing significant difference of serum TK activity in the studied age groups (D).

In the healthy cohort, there was no significant difference in TK1 activity between the men and women studied, as such gender was not taken into consideration when defining age groups. TK1 activities of 3 age groups are shown on Figure 2A-C. There was a significant difference between the TK1 activity of the young (n = 95) and the elderly (n = 83), (11.85±5.7 U/L vs. 8.65±3.9 U/L; p<0.001) and between the young and the middle-aged (n = 191) (11.85±5.7 U/L vs. 9.79±5.2 U/L; p<0.001). The difference was at the limit of statistical significance between the middle-aged and the elderly (9.79±5.2 U/L vs. 8.65±3.9 U/L; p = 0.050). Overall, the lowest observed TK1 level was 0.5 U/L and the highest was 35.6 U/L. The 97.5 percentiles in the 3 age groups were 29.8 U/L (young), 25.4 U/L (middle-aged) and 19 U/L (elderly) (Figure 2D).

**Figure 3:**
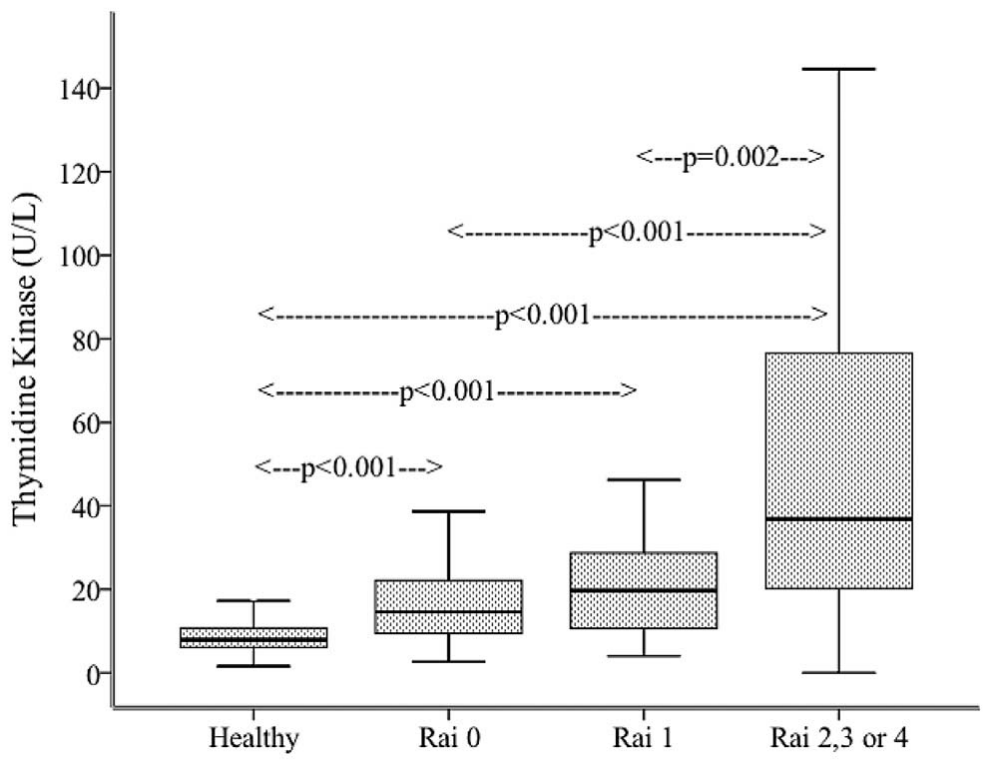
Comparison of serum thymidine kinase activity in de novo CLL patients in Rai 0, Rai 1 and Rai 2-3-4 clinical stages with the age and sex matched healthy cohort.

Results of 115 CLL patients on presentation were compared with an equal number of age- and sex-matched controls (n = 115). This age- and sex-matched control group was derived from the over-all healthy cohort (n = 393), that was used to define the reference ranges of TK1 in the different age groups. Patient characteristics are given in Table 2. We found significant difference in TK1 activity between the healthy control group and all Rai stages of CLL. As we had few individuals in Rai 2, Rai 3 and Rai 4 stages, we grouped them as being in a more advanced stage of the disease. The observed mean TK1 activity in the age and sex matched healthy group (n = 115) was 9.18±5.0 U/L, which was significantly lower than that in Rai 0 (n = 43; mean: 18.58±15.6 U/L; p<0.001), Rai 1 (n = 27; mean: 28.16±32.9 U/L; p<0.001) and Rai 2-3-4 (n = 45; mean: 72.88±101.1 U/L; p<0.001). We also noticed significant difference in TK1 activity between Rai 0 and Rai 2-3-4 stages (p<0.001) and Rai 1 and Rai 2-3-4 stages (p = 0.002) (Figure 3).

**Table 2:** CLL patient characteristics.

	CLL patients (n = 115)
Age, years (mean, range)	65.6 (46–86)
Males (n, %)	61 (53)
Females (n, %)	54 (47)
Rai 0 (n, %)	43 (37)
Rai 1 (n, %)	27 (23)
Rai 2 (n, %)	19 (17)
Rai 3 (n, %)	15 (13)
Rai 4 (n, %)	11 (10)
CD38–/Zap70– (n, %)	52 (45)
CD38–/Zap70+ (n,%)	17 (15)
CD38+/Zap70– (n, %)	15 (13)
CD38+/Zap70+ (n, %)	31 (27)
White blood cell count, G/L (mean, range)	50.6 (12.4–447)
Absolute B cell count, G/L (mean, range)	39.9 (5.2–392)

CLL patients were on average 65.6 years old (range: 46–86). There was no difference in TK1 levels upon comparing those below and equal to 60 years (middle aged, n = 29) and those over 60 years (elderly, n = 86) of age belonging to the different Rai stages (Table 3).

**Table 3:** Comparison of thymidine kinase activity between middle-aged and elderly CLL patients belonging to different Rai stages.

Rai stages	Age		p values
	46–60 years	60.1–86 years	
	Thymidine kinase activity, mean ± SD (U/L)		
Rai 0 (n = 43)	15.5±8.9 (n = 15)	20.2±18.2 (n = 28)	0.558
Rai 1 (n = 27)	59.7±67.7 (n = 5)	21.0±13.3 (n = 22)	0.524
Rai 2, 3 or 4 (n = 45)	89.0±151.6 (n = 9)	68.8±86.7 (n = 36)	0.645

**Figure 4:**
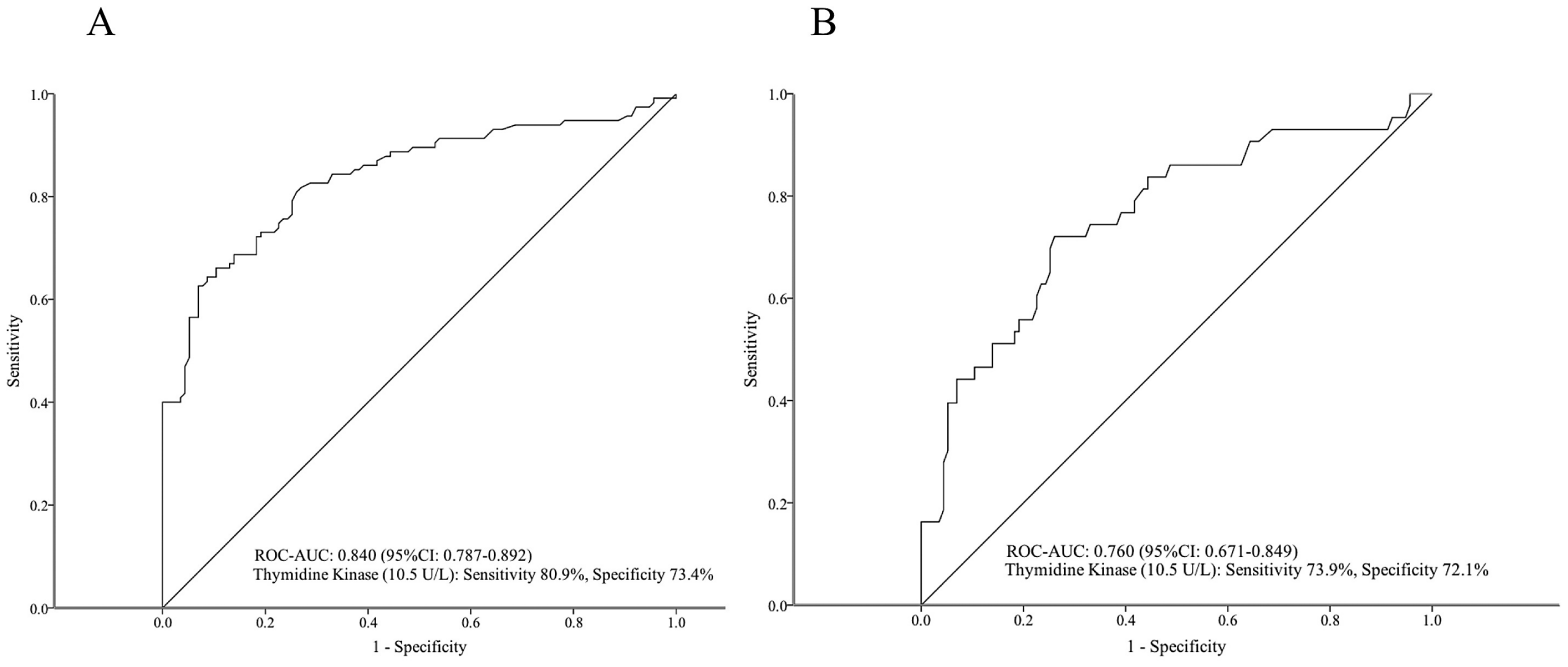
Receiver operating characteristic (ROC) analysis of serum thymidine kinase activity for differentiating all CLL patients (A) and Rai 0 CLL patients (B) from age and sex matched healthy controls. AUC, area under the receiver operating characteristic curve.

The area under the receiver operating characteristic curve (ROC-AUC) for TK1 was 0.840 (95% CI: 0.787–0.892) for differentiating CLL patients from age and sex matched healthy controls, with a cut-off value of 10.5 U/L (sensitivity: 80.9%, specificity: 73.4%) (Figure 4A.). Furthermore, at the same cut-off value, the ROC-AUC for TK1 was 0.760 (95% CI: 0.671–0.849) for differentiating Rai 0 stage CLL patients from healthy controls (sensitivity: 73.9%, specificity: 72.1%) (Figure 4B.).

**Figure 5:**
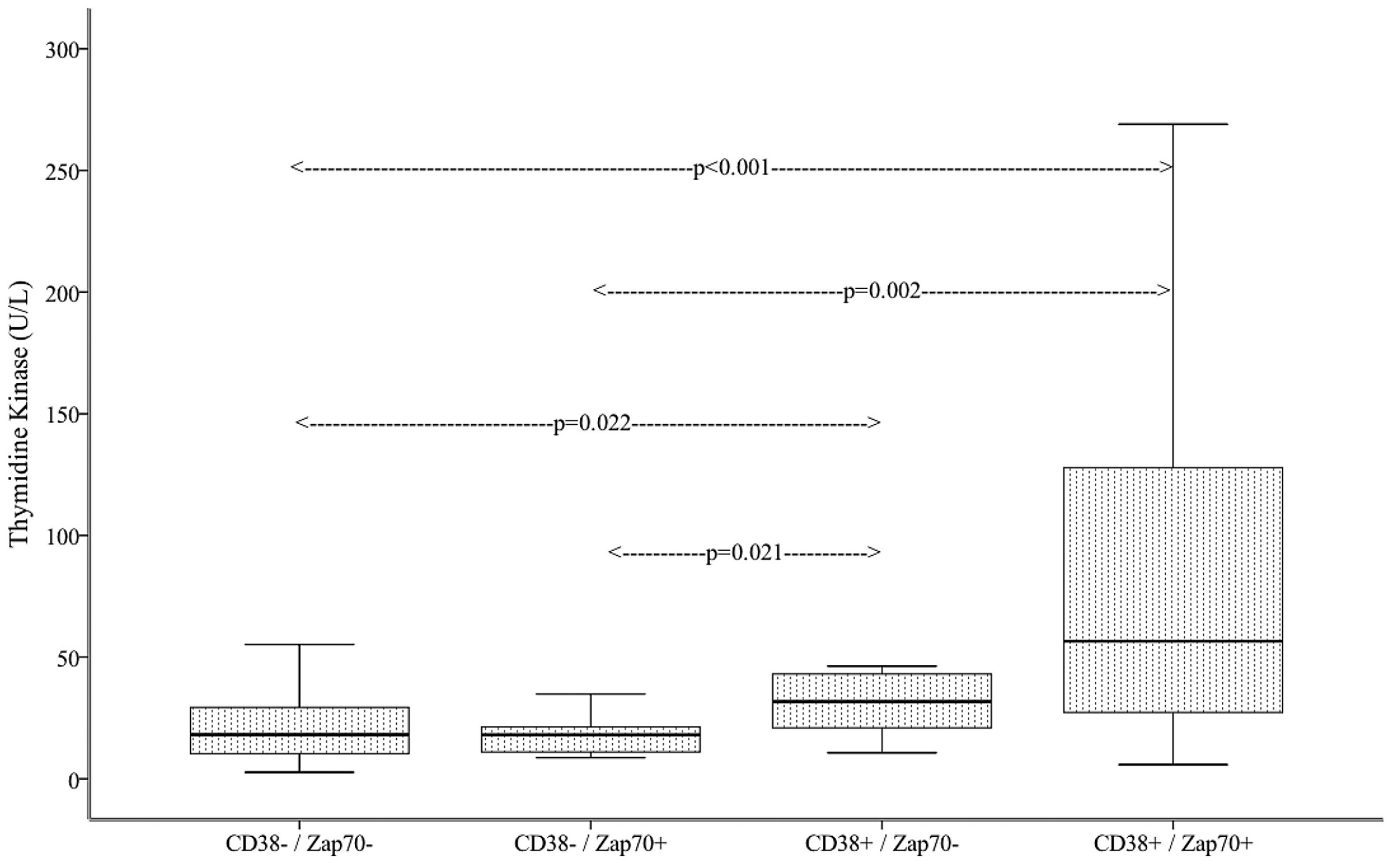
Serum thymidine kinase activity of CLL patients upon presentation in different prognostic groups.

We also compared the TK1 results of the CLL patients with other known prognostic factors, i.e., CD38 and Zap70 expression. The mean TK1 activity in the CD38–/Zap70– group (n = 52) was significantly lower than that in the CD38+/Zap70+ group (n = 31) (22.3±17.8 U/L vs. 109±130.3 U/L; p<0.001). The difference was also significant between the CD38–/Zap70+ group (n = 17) and the CD38+/Zap70+ group (26.25±33 U/L vs. 109±130.3 U/L; p = 0.002); and between the CD38+/Zap70– group (n = 15) and the CD38–/Zap70– group (40.87±38 U/L vs. 22.3±17.8 U/L; p = 0.022); and the CD38–/Zap70+ group and the CD38+/Zap70– group (26.25±33 U/L vs. 40.87±38 U/L; p = 0.021) (Figure 5.).

With ROC analysis, the TK1 cut-off value to distinguish CD38–/Zap70– patients from those with either or both positivity was 30 U/L (ROC-AUC: 0.710, 95%CI: 0.601–0.819, Specificity:76.6%, Sensitivity: 54.3%).

Out of 115 CLL patients, 95 had FISH data. Upon comparing those with no chromosomal alterations, del(13q), 12 trisomy (n = 76) with the group with del(17p13), del(11q22) (n = 19) no statistically significant difference was found in the TK1 values (38.7±65.7 U/L vs. 55.5±96.5 U/L, p  =  0.347). The non-significance did not change when patients with 12 trisomy were excluded from the statistical analysis (33.0±44.5 vs. 55.5±96.5, p  =  0.211).

We observed a statistically significant correlation between TK1 activity and WBC (Spearman’s rho: 0.367, p<0.001) and absolute B cell count (Spearman’s rho: 0.369, p<0.001).

## Discussion

According to the 2008 guideline of the Clinical Laboratory Standards Institute, it may be necessary to carry out a minor reference value study to validate a reference interval given by the test manufacturer or a donor laboratory [37]. Soon after we started our TK1 measurements with the test manufacturer’s reference range (2 – 7.5 U/L) it turned out that it was not applicable to our population, as we often observed higher TK1 activity than the suggested upper reference limit even in healthy individuals. The interpretation of these results was not always convincing and caused some unnecessary anxiety for physicians and patients. The observed elevated TK1 activity in our healthy population and the significant differences in TK1 activity between the age groups encouraged us to define our own reference ranges for TK1. Although TK1 measurements are widely used in malignant diseases for prognostic purposes and even in routine health screening [38], to our knowledge no age-related reference ranges have been published so far for TK1 activity.

In each age group we had sufficient numbers of reference subjects to determine the 97.5 percentiles. Based on our 97.5 percentile results, the suggested reference values are <30 U/L for young (18–35 years), <25 U/L for middle-aged (36–60 years) and <19 U/L for elderly (60–86 years) adults. The age-specific reference ranges may be crucial in the context of chronic lymphocytic leukemia, where most of the affected are older individuals. We also identified a cutoff point of 10.5 U/L distinguishing CLL patients in the RAI 0 stage from healthy individuals. This result correlates well with the finding of Letestu et al, who found a serum TK1 activity of 10 U/L to be an independent predictor of progression-free survival with a hazard ratio of 2.98, p<0.0001 [39].

As TK1 is a general proliferation marker, several non-malignant conditions like infection, inflammation, autoimmune disorders and benign tissue proliferations might also cause mildly elevated TK1 levels [40]. Hagberg et al showed that severe vitamin B12 deficiency caused elevated serum TK1 levels, as the proliferating tissues became unstable in the absence of the vitamin [41]. Chen et al also showed that serum TK1 concentration was useful in health screening as their patients with elevated TK1 concentration had a significantly increased risk of developing malignancies [42]. The above study also showed that a significantly larger proportion of oilfield workers had elevated serum TK1 concentrations than city dwellers [42]. As such, in routine medical practice, non-malignant medical conditions and environmental factors need to be considered when using TK1 as a health screening marker.

It is also known that CD38+ CLL has a more adverse prognosis [43]. Hus et al showed that CD38 combined with Zap70 expression amplified the prognostic power of both markers. They found that Zap70+/CD38+ patients had shorter event-free survival than CD38–/Zap70– patients [44]. In our study, we found significantly higher TK1 activity in CD38+/Zap70+ as compared to CD38–/Zap70– patients and there was no significant difference between CD38–/Zap70– and CD38–/Zap70+ patients. Higher serum TK1 activities of CD38+ CLL patients can be explained by the intense proliferation of cells, as both TK1 and CD38 are proliferation markers [45], [35].

Furthermore, in line with published data, del(13q), 12 trisomy, del(17p13) and del(11q22) FISH aberrations did not influence TK1 values in our patients [16].

TK1 activity correlated with WBC and absolute B cell count in our study, this finding supports the data reported previously by Hallek et al [15].

Each laboratory should establish its own age-related reference ranges as even minor ethnical or lifestyle differences in a particular region may have a significant impact on reference ranges, not disregarding the influence of age and sex. These differences make it difficult to obtain universally applicable reference intervals, even though the applied methodology is traceable to reference measurement systems [46], [47]. As producing reference ranges is an expensive task for an individual laboratory, one possible solution is to establish reference ranges for a homogeneous population by involving all the laboratories measuring the certain analyte with the same method [47]. Siest et al emphasized that the boundaries between healthy and pathological conditions are not clear-cut, and reference intervals or cut-off values should be established to expedite clinical decision making [48].

Limitations of the present results include the cross-sectional nature of the CLL study, a prospective design would have enabled us to examine the utility of TK1 as a prognostic marker. Although the CLL population was represented by a cohort of 115 de novo patients, the non-similar number of patients in the different Rai stages, and as per their CD38 and Zap70 characteristics may have distorted the statistical findings. Furthermore, our age-specific reference ranges need to be validated in other healthy populations as well.

In conclusion, serum TK1 levels decline significantly with age in the healthy, and this finding warrants the use of age-related reference ranges. Furthermore, it may distinguish CLL patients in the RAI 0 stage from healthy individuals in the diagnostic work-up of CLL suspect cases.
